# Gain- and Loss-Related Brain Activation Are Associated with Information Search Differences in Risky Gambles: An fMRI and Eye-Tracking Study

**DOI:** 10.1523/ENEURO.0189-16.2016

**Published:** 2016-09-22

**Authors:** Alexander Niklas Häusler, Sergio Oroz Artigas, Peter Trautner, Bernd Weber

**Affiliations:** 1 Center for Economics and Neuroscience, University of Bonn, 53127 Bonn, Germany; 2 Department of Epileptology, University Hospital Bonn, 53127 Bonn, Germany; 3 Department of NeuroCognition/Imaging, Life & Brain GmbH, 53127 Bonn, Germany; 4 Department of Psychology, University of Lübeck, 23562 Lübeck, Germany

**Keywords:** decision-making, eye-tracking, fMRI, information search, reward

## Abstract

People differ in the way they approach and handle choices with unsure outcomes. In this study, we demonstrate that individual differences in the neural processing of gains and losses relates to attentional differences in the way individuals search for information in gambles. Fifty subjects participated in two independent experiments. Participants first completed an fMRI experiment involving financial gains and losses. Subsequently, they performed an eye-tracking experiment on binary choices between risky gambles, each displaying monetary outcomes and their respective probabilities. We find that individual differences in gain and loss processing relate to attention distribution. Individuals with a stronger reaction to gains in the ventromedial prefrontal cortex paid more attention to monetary amounts, while a stronger reaction in the ventral striatum to losses was correlated with an increased attention to probabilities. Reaction in the posterior cingulate cortex to losses was also found to correlate with an increased attention to probabilities. Our data show that individual differences in brain activity and differences in information search processes are closely linked.

## Significance Statement

The processing of gains and losses has been thoroughly investigated in the field of decision-making using different methods, such as eye tracking and neuroimaging. Even though previous studies have combined both of these methods in single tasks before, this is the first study that correlates the results from two separate tasks using either method. Using this approach, we show for the first time that individual differences in neural gain and loss processing relate to individual differences in the information search phase of risky gambles. These results emphasize the functional interplay between attention and the neural circuits of reward and loss processing.

## Introduction

When individuals are confronted with risky decisions, they have to choose between options that entail different outcomes with known probabilities of realization. Risk-averse individuals prefer a safe over a risky gamble of equal expected value (EV), with the opposite being true for risk-seeking individuals. Even though risk preferences are mostly investigated using self-assessments or behavioral tasks in an experimental setting, they have been shown to relate to important real-life social and economic outcomes. Risk-seeking individuals are, for example, more likely to migrate (Jaeger et al., 2010) and to have higher-earning occupations (Bonin et al., 2007). Since attention differences measured via eye tracking (Brandstätter and Körner, 2014) and individual differences in neural processing of risk (Rudorf et al., 2012) have both been shown to relate to risk preferences, the investigation of the relation between attention and neural processing is an important and necessary step to enhance our understanding of human decision-making under risk.

Functional magnetic resonance imaging (fMRI) and eye tracking have widely been used to investigate the neural correlates and behavioral aspects of decision-making under risk. Recently, an “affect-integration-motivation” framework has been presented as a model that integrates the affect, integration, and motivational aspects of decisions that involve gains, losses, and risks (Samanez-Larkin and Knutson, 2015). This cognitive-processing framework is based on several studies that unveil the neural circuits involved in the processing of rewards, losses, and risks. One of these brain areas is the ventral striatum (VS), which has long been known as a key region in reward and risk processing (Schultz et al., 1997; Knutson et al., 2000; Kuhnen and Knutson, 2005; Fliessbach et al., 2010; Bartra et al., 2013; Clithero and Rangel, 2014; Samanez-Larkin and Knutson, 2015). The ventromedial prefrontal cortex (vmPFC) is another region that has been found to play a role in reward processing through its role in valuation (Fliessbach et al., 2010; Bartra et al., 2013; Clithero and Rangel, 2014). Both regions have also been related to processing losses (Seymour et al., 2007; Tom et al., 2007; Cooper and Knutson, 2008), which has been interpreted as a representation of a gain–loss continuum (Tom et al., 2007). Besides these two areas, the anterior insula (AI) has been shown to play a major role in loss processing (Samanez-Larkin et al., 2008; Fukunaga et al., 2012), with its activation also preceding risk-averse choices (Kuhnen and Knutson, 2005). Even though it has been shown that many different cortical and subcortical regions are involved in processing positive and negative outcomes (Vickery et al., 2011), we are focusing on the three mentioned brain regions, because the individual activation differences in the VS and AI have been related to risk preferences and even to financial success in a stock market experiment (Samanez-Larkin et al., 2008; Rudorf et al., 2012; Smith et al., 2014) and the vmPFC has been robustly linked to valuation (Bartra et al., 2013; Clithero and Rangel, 2014).

Behavioral results have noted for a long time the tendency of individuals to place more weight on outcomes compared with probabilities (Daston, 1995; Arnauld and Nicole, 1996; Loewenstein et al., 2001; Sunstein, 2003). More recently, studies involving eye tracking have been aiming to dig deeper into the underlying causes of such tendencies by measuring information search processes in risky gambles. Information search processes are behaviorally expressed through eye movements that can be traced and recorded. Especially attention, measured through the number of fixations, has been studied in this context, and attention differences to values and probabilities of risky choices in both the gain and loss domain have been found (Brandstätter and Körner, 2014). Even though individual differences in attention have been shown (Fiedler and Glöckner, 2012), it is unclear up to now whether and how they relate to individual differences in neural gain and loss processing.

Taking into account the findings from each of these studies using different techniques, we propose that information search in risky choices is related to the neural processing of gains and losses. Using two independent experiments that involve fMRI and eye tracking, we describe the link between individual attention differences and activation during reward and loss processing in the VS, vmPFC, and AI.

## Materials and Methods

Over the course of 2 months, 50 healthy adult males (25.9 ± 4.55 years) participated in a study consisting of two independent parts measured on the same day: an fMRI and an eye-tracking session. Exclusion criteria were a history of neurological or psychiatric disorders, conditions prohibiting the participation in an MRI setting, and imperfect eyesight. Upon arrival and prior to the tasks, a thorough instruction was handed out, explained, and discussed. The study was approved by the Ethics Committee of the University of Bonn, and all subjects gave written informed consent according to the Declaration of Helsinki (World Medical Association, 2004).

### fMRI acquisition and paradigm

**Figure 1.**
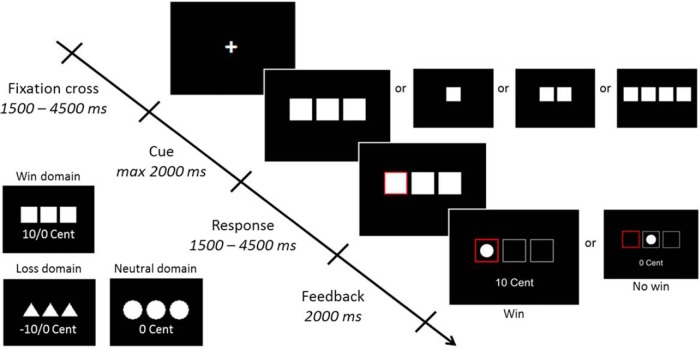
The fMRI paradigm timeline and symbol explanation for each of the three domains. Each subject completed 48 win, 48 loss, and 24 neutral trials, and the symbol–domain relationship was counterbalanced.

Participants underwent a structural T1 measurement (160 slices; voxel size, 1 × 1 × 1 mm; repetition time (TR), 1660 ms; echo time (TE), 3.09 ms; and flip angle, 15°) in a 1.5 T Avanto Magnetom scanner (Siemens) using a standard eight-channel matrix head coil. Afterward, participants completed an fMRI paradigm (Fig. 1), which was an extended version of a previously established choice task (Fliessbach et al., 2010; Rohe et al., 2012; Häusler et al., 2015). In this task, participants were asked to guess under which of one, two, three, or four symbols a ball was hidden (Fig. 1). Our task was thus related to the popular shell game, with the difference being that ours was a pure guessing task excluding deception and including varying probabilities of guessing correctly due to the varying number of symbols (e.g., 100% in case of one symbol, 50% for two symbols). The nondeception aspect was made especially clear to the participants to avoid inconsistent brain responses due to possible biases coming from deception experienced during observation of the real-life shell game, in which participants are often deceived. Furthermore, whereas the original paradigm used in previous studies was composed of situations with different probabilities in only the win domain, the new paradigm was adapted to also involve monetary loss and neutral situations. The paradigm consisted of 120 total trials: 48 in the win, 48 in the loss, and 24 in the neutral domain. Each of the three domains (win, loss, and neutral) was represented by a different symbol, namely squares, triangles, and circles (Fig. 1). The mapping between a domain and its specific cue symbol was counterbalanced across subjects. The sequence of trials was randomized with the condition in order for a trial of a specific domain to not be followed by a trial of the same domain. The paradigm was programmed using in-house software based on Python (version 3.4; RRID:SCR_008394). Images were displayed via video goggles, and participants made decisions via response grips (both from Nordic NeuroLab) using the index fingers and thumbs of both hands.

First, a fixation cross was shown with a randomized duration between 1500 and 4500 ms. In the second phase (the cue phase), each participant saw one, two, three, or four symbols, all from the same domain (either win, loss, or neutral). Subjects were told that selecting one specific symbol would lead to a win, a loss, or nothing, depending on the domain. Subjects had up to 2000 ms to choose the respective target symbol. The number of items (one, two, three, or four) were shown next to each other and represented the chances of winning (reward probability: 100%, 50%, 33%, and 25%) or losing (loss probability: 0%, 50%, 66%, and 75%) 10 € cents. Guessing incorrectly in the win domain led to no win, and guessing incorrectly in the loss domain led to a loss of 10 € cents. The participants did not win or lose any money in the neutral domain.

After pressing one of the four buttons, the selected option was highlighted for a randomized time between 1500 and 4500 ms. Last, the result was presented in an outcome feedback phase during which the participants found out whether they won or lost 10 € cents, or did not win or lose any money. Functional data were acquired using a TR of 2.5 s, a TE of 45 ms, and a flip angle of 90º.

Each volume contained 31 slices with a voxel size of 3 × 3 × 3 mm, covering the whole brain, including midbrain but sparing part of the cerebellum. A total of 800 scans were acquired. At the end of the scanning session, each subject was informed about the total amount of money won (outcome of each task plus a 15 € participation fee) during the first part and that this monetary win was independent of subsequent results in the eye-tracking session.

### Eye-tracking acquisition and paradigm

The participants took a 5–10 min break between both experiments in the non-laboratory-related waiting room of our institute. They were then accompanied to the eye-tracking laboratory and asked to sit comfortably while resting the head on a chinrest. They were instructed to make a total of 80 decisions while undergoing eye-tracking recordings from the left eye at 1000 Hz using an Eyelink 1000 eye-tracker (SR Research; RRID:SCR_009602). The eye-tracking experiment was programmed using in-house software based on Python 3.4, and each participant completed a nine-point calibration and a practice phase before starting with the experimental trials.

**Figure 2.**
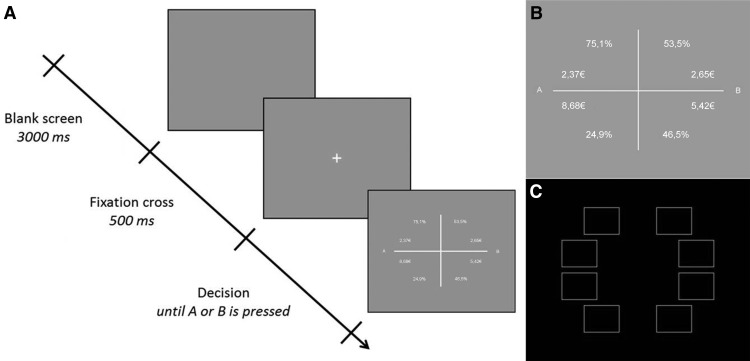
The eye-tracking experiment. ***A***, Paradigm timeline. ***B***, Exact display of the eye-tracking stimulus shown during the decision phase. ***C***, The eight areas of interest used to extract the number of fixations.

Each trial consisted of a blank screen (3000 ms) to rest the eye, a fixation cross (500 ms), and a decision phase with no time limit showing two lotteries (Fig. 2*A*). The participants were asked to opt for lottery A or B during the decision phase, as indicated by the respective letters “A” and “B” positioned next to the two lotteries (Fig. 2*B*). White horizontal and vertical lines were used to divide the lottery options in order to make the decision process more intuitive for the participants. The participants were then able to press one of two keyboard letters to enter their decisions for options A or B, respectively. In each lottery (Fig. 2*B*, lottery A), two different monetary amounts were presented as possible outcomes to the subject. Both amounts of money were associated with respective probabilities, and the number of digits was identical for both amounts and percentages. The stimuli were shown as white writing on gray background, a color scheme used in a previous eye-tracking study (Fiedler et al., 2013). The areas of interest (AOIs; size, 200 × 150 pixels) centers were positioned at the same distance from the total image center (Fig. 2*C*).

A script in MATLAB R2014a (MathWorks; RRID:SCR_001622) was used to create gambles in which the domain, winning/losing probabilities, and winning/losing values were pseudo-randomized. Different ranges of values (Vs; Fig. 2) and probabilities (Ps) were used to create a high-risk and a low-risk lottery. The EVs for both the high-risk and the low-risk options in our experiment were chosen to be within a range of 3–5 € and always similar between the two lotteries. This was done because a previous study by Fiedler and Glöckner (2012) found the decision time and the number of fixations to increase with mean EV. The riskiness of a lottery was defined as the difference between the two monetary amounts (i.e. the possible variance of the outcomes). In Figure 2, the high-risk lottery would therefore be “A” and the low-risk lottery would be “B,” even though the EV of both gambles is 3.94 €. The locations of the two lotteries were randomized between left and right, and the locations of the values and probabilities (upper or lower) varied between subjects and were counterbalanced.

For each domain, 30 value and probability combinations were pseudorandomly chosen from a list of 175,000 generated combinations containing all of the mentioned parameters. None of the probabilities were used more than once. Additionally, 10 distractors for each domain (random values and probabilities with the same amount of digits) were generated in order to mix up the paradigm and thus require each subject to concentrate in every trial. Hence, each domain (win and loss) contained 30 experimental and 10 distractor trials, summing up to the total of 80 trials. At the end of the experiment, one trial from each domain was randomly selected and paid to the participant on top of the participation fee (15 €). This amount was added to the amount won in the first part of the experiment and transferred to the participant’s bank account.

### fMRI analysis

Datasets of two participants were excluded due to both participants not having understood the fMRI task correctly. None of the participants exceeded head motion limits (translational, >3 mm; rotational, >2.5°), thus leading to an fMRI analysis of 48 participants. The fMRI analysis was performed using Statistical Parametric Mapping software version 12 (SPM12, Wellcome Department of Imaging Neuroscience; RRID:SCR_007037) through scripts written in MATLAB. Preprocessing included slice time correction, motion correction, spatial normalization to the canonical template from the Montreal Neurological Institute (MNI), reslicing to a 3 × 3 × 3 mm voxel size, and spatial smoothing using a Gaussian kernel with full-width at half-maximum of 8 mm. In the first-level analysis, a general linear model (GLM) was created (Table 1) with the aim of analyzing the prediction and prediction error in both the reward and loss domain [reward prediction (RP), loss prediction (LP), reward prediction error (RPE), and loss prediction error (LPE); Table 2]. The following four parametrical contrasts were defined: “RP > 0” and “LP > 0” (both at anticipation phase, Table 1), as well as “RPE > 0” and “LPE > 0” (both at feedback phase, Table 1). The first-level contrasts were used for the second-level analysis.

**Table 1:** Overview of the GLM used for estimating brain activation

Regressor	Parametrical modulation	Contrasts of interest
Onset of choice, win domain	Yes: RP	RP > 0
Onset of choice, loss domain	Yes: LP	LP > 0
Onset of choice, neutral domain	Yes: RP	
Onset of result, win domain	Yes: RPE	RPE > 0
Onset of result, loss domain	Yes: LPE	LPE > 0
Onset of result, neutral domain	Yes: RPE	
Six movement regressors	NA	

NA, not applicable.

**Table 2:** Overview of the parametric modulator calculations used for estimating brain activation

	Number of symbols shown	RP	RPE in case of win (1): RPE = 1 − RP	RPE in case of no win (0): RPE = 0 − RP
Win domain	1	1	1 − 1 = 0	0 − 1 = −1
	2	1/2	1 − 1/2 = 1/2	0 − 1/2 = −1/2
	3	1/3	1 − 1/3 = 2/3	0 − 1/3 = −1/3
	4	1/4	1 − 1/4 = 3/4	0 − 1/4 = −1/4
	Number of symbols shown	LP	LPE in case of loss: LPE = −1 − LP	LPE in case of no loss: LPE = 0 − LP
Loss domain	1	0	−1 − 0 = −1	0 − 0 = 0
	2	−1/2	−1 − (−1/2) = −1/2	0 − (−1/2) = 1/2
	3	−2/3	−1 − (-2/3) = −1/3	0 − (−2/3) = 2/3
	4	−3/4	−1 − (−3/4) = −1/4	0 − (−3/4) = 3/4

In order to investigate individual differences and relate brain activity to behavioral and eye-tracking measures, activations from three independent and previously defined 6 mm spherical regions of interest (ROIs) were used. These included the VS and AI using coordinates from a recent study by Smith et al. [2014; MNI coordinates (*x*, *y*, *z*): AI, ±36, 24, 2; VS, ±12, 8, −8]. Additionally, vmPFC coordinates were obtained in a manner similar to that of Smith et al. (2014) by entering the brain term (“ventromedial prefrontal”) into the “Neurosynth.org” database (accessed on February 17, 2016) and obtaining the peak MNI coordinates [of 250 studies (*x*, *y*, *z*): ±4, 42, −8]. We additionally included the oral area of the somatosensory cortex (OSS; *x*, *y*, *z*: ±64, −13, 14; Miyamoto et al., 2006).

The AFNI (Analysis of Functional Neuroimages; RRID:SCR_005927) program 3dClustSim, which is based on Monte Carlo simulations, was used to obtain cluster-size threshold information to correct for multiple comparisons (http://afni.nimh.nih.gov/pub/dist/doc/program_help/3dClustSim.html). After observing that the posterior cingulate cortex (PCC) was also associated with reward processing in our experiment, we decided to include this activation cluster in an explorative analysis. An ROI mask of the PCC was created using the second-level contrast “RPE > 0” at a cluster size FWE-corrected *p* value of 0.05 (MNI coordinates *x*, *y*, *z*: 0, −16, 44). Beta values from all of the ROIs were extracted using the MarsBaR (MARSeille Boîte À Région d’Intérêt, RRID:SCR_009605) ROI toolbox for SPM (Brett et al., 2002).

### Eye-tracking analysis

Datasets of six participants had to be excluded. These exclusions arose due to the loss of one dataset, unfeasible calibrations of four participants, and one participant having fixated only one of the options in too many trials (±2 SDs outside of the mean). Eye-tracking fixations of the remaining 44 participants were furthermore checked for a gaze stability of at least 50 ms, with fixations <50 ms subsequently being excluded. Data viewing, and the corresponding fixation extraction for each of the AOIs was performed using the Eyelink Data Viewer version 1.10 (SR Research), while reaction times and choice results were extracted using in-house software based on Python version 3.4. Descriptive overviews were performed using IBM SPSS Statistics 22 (IBM; RRID:SCR_002865). Correlation analysis of only the eye-tracking data was performed using Pearson correlations in STATA version 13 (StataCorp LP; RRID:SCR_012763).

### Correlation analysis of fMRI and eye-tracking data

After previous exclusions of both eye-tracking and fMRI data, analyses of the remaining 43 datasets were performed using STATA version 13. To test our initial hypotheses of brain activation correlating with attention patterns, we created the two variables “Df win” [difference in fixations between values (*f*[*v*]) and probabilities (*f*[*p*]) in the win domain] and “Df loss” [difference in fixations between values (*f*[*v*]) and probabilities (*f*[*p*]) in the loss domain].

Both are defined as the difference of fixations between values and probabilities in such a way that a positive value reflects more fixations on values compared with probabilities. Additionally, the variable “Df high risk” for both the win and the loss domain represent the difference in fixations between the high-risk [*f*(*h*)] and low-risk [*f*(*l*)] lotteries, with a positive value reflecting more fixations on the high-risk versus the low-risk gambles. After creating these variables, all extracted fMRI β values from the gain and loss domain were correlated with Df win and Df loss, respectively. These estimated Pearson correlations were subsequently bootstrapped (seed set at 10; repetitions, 10,000) and reported.

## Results

### Whole-brain fMRI

**Figure 3.**
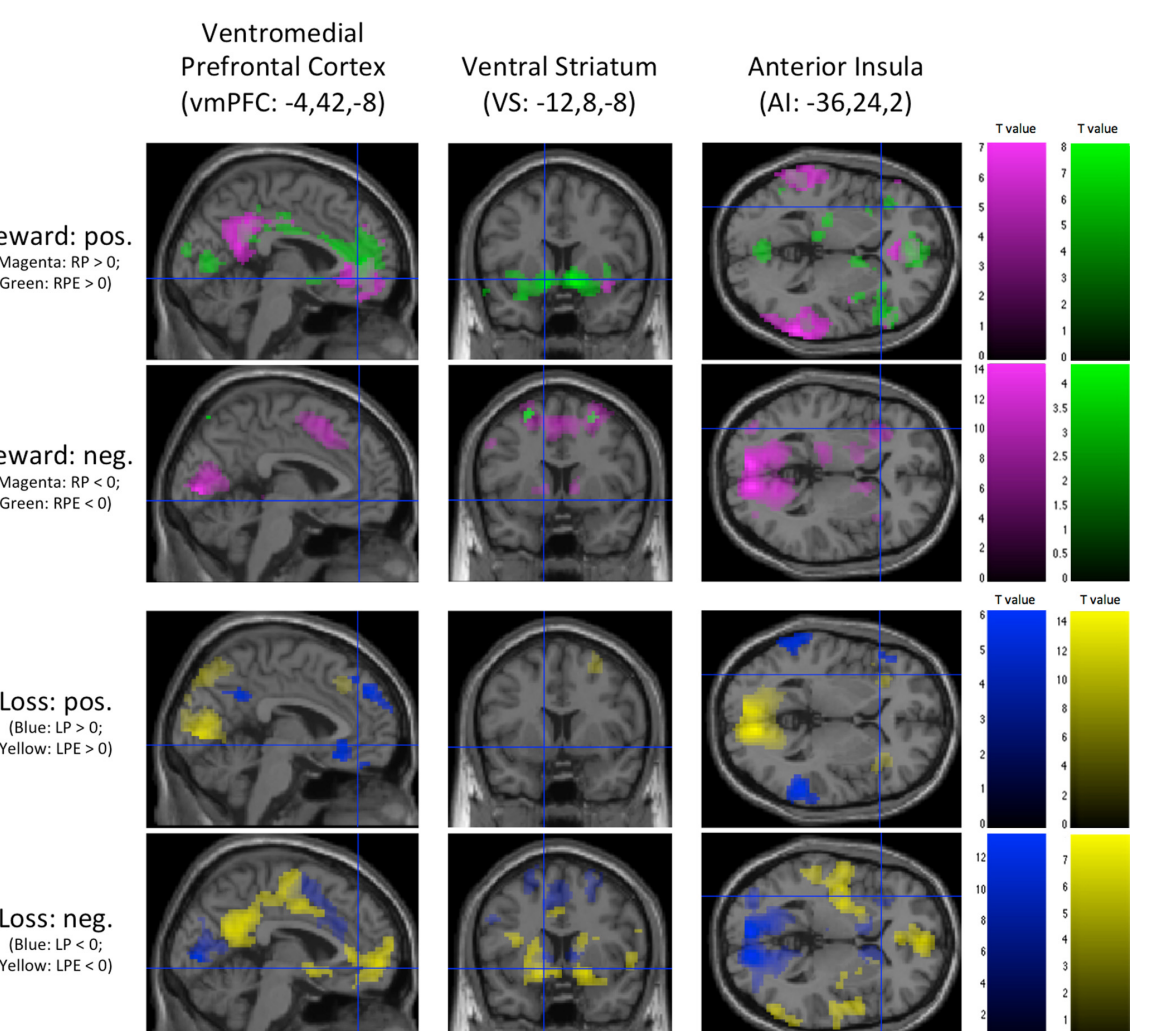
Whole-brain activation during the different parts of reward and loss processing (whole-brain corrected *p* < 0.05, based on 3dClustSim correction [*k* > 33, *p* < 0.005], df = 47) and obtained using the fMRI paradigm. Color coding: magenta, RP; green, RPE; blue, LP; yellow, LPE. The first and the third row represent the positive contrasts vs baseline (>0), and the second and fourth row represent the negative contrasts vs baseline (<0) in the reward and loss domains, respectively. Respective *t* value color bars are shown on the right side.

Brain regions corresponding to the reward and loss processing cluster peaks shown in Tables 3 and 4 are reported in the following two paragraphs and can also be seen in Figure 3. The reported activations are thresholded at a cluster size FWE-corrected *p* value of 0.05.

**Table 3:** Whole-brain activity related to RP and RPE

Contrast	Region	Laterality	MNI coordinates			Cluster size	*t*	Cluster *P* (FWE corrected)
			*x*	*y*	*z*			
RP > 0	MTG	R	66	−49	2	1368	7.01	<0.001
	oMFG	L	−57	29	−10	2017	6.70	<0.001
	vmPFC	L	−9	38	−7	2017	5.64	<0.001
	MTG	L	−63	−55	29	1337	6.17	<0.001
	PCUN	L	3	−52	20	671	6.07	<0.001
	POG	R	39	−19	56	175	5.05	0.011
RPE > 0	VS	L	−12	5	−10	1805	7.95	<0.001
	VS	R	12	5	−10	1805	7.84	<0.001
	vmPFC	L	−6	44	−1	1094	6.87	<0.001
	vmPFC	R	3	47	−1	1094	6.35	<0.001
	MTG	L	−60	−46	5	189	4.35	0.004
	VIS	L	−3	−76	2	235	4.33	0.001
	MCC	L/R	0	−16	44	317	4.26	<0.001

Cluster size FWE-corrected, voxel threshold = 0.005; df = 47. L, Left; R, right.

**Table 4:** Whole-brain activity related to LP and LPE

Contrast	Region	Laterality	MNI coordinates			Cluster size	*t*	Cluster *p* (FWE corrected)
			*x*	*y*	*z*			
LP > 0	dmPFC	R	3	50	35	392	6.06	<0.001
	TPJ	L	−57	−67	29	647	5.76	<0.001
	MTG	R	60	−43	5	159	5.68	0.015
	PCUN	R	6	−55	35	139	5.19	0.028
	MTG	L	−63	46	−1	174	4.90	0.001
	TPJ	R	57	−58	35	447	4.68	<0.001
	vmPFC	L/R	0	26	−16	158	4.49	0.015
LPE > 0	VIS	R	9	−79	−1	4845	14.54	<0.001
	AI	R	42	20	−1	129	5.47	0.038

Cluster size FWE-corrected, voxel threshold = 0.005, df = 47. L, Left; R, right.

#### Reward domain

 The bilateral middle temporal gyrus (MTG), orbital part of the left middle frontal gyrus (oMFG), vmPFC as well as the left precuneus (PCUN), and right postcentral gyrus (POG) were all activated during the reward anticipation phase with increasing reward prediction (RP > 0; Table 3; Fig. 3, magenta, first row). The PCUN cluster also included activation in the midcingulate cortex (MCC) and the PCC. The reward prediction error parameter (RPE > 0) correlated, among others, with activity in the bilateral VS, vmPFC, and MCC (Table 3; Fig. 3, green, first row). Notably, a large portion of the MCC cluster was located in the PCC.

#### Loss domain

During the loss anticipation phase (LP > 0), the temporal parietal junction (TPJ), MTG, and vmPFC were all activated bilaterally (Table 4; Fig. 3, blue, third row). Additionally, the right PCUN and dorsomedial prefrontal cortex (dmPFC) were activated as well. Brain areas correlating with the loss prediction error parameter (LPE > 0) included the visual cortex (VIS) and the AI (Table 4; Fig. 3, yellow, third row).

### ROI fMRI

In the following paragraph, we report brain activation in three a priori determined ROIs: the vmPFC, VS, and AI. The activations sustained a whole-brain correction of 0.05 and custom 3dClustSim thresholds (Tables 5, 6). Activation in these regions is also shown as part of the whole-brain fMRI activation depicted in Figure 3. The reward prediction parameter “RP > 0” correlated with activity in the vmPFC (Table 5), while the opposite contrast only showed activation of the left AI (Table 5). Investigating activity correlating with the RPE during the outcome phase resulted in bilateral activation in all of the ROIs (Table 5), while the opposite contrast showed no significant activation (Table 5). Contrasting loss prediction to baseline, we observed the bilateral vmPFC to be activated (Table 6), while the opposite contrast resulted in activation of the left AI and the bilateral VS (Table 6). Only the bilateral AI was correlated positively with the LPE parameter (Table 6), while the bilateral vmPFC and VS correlated negatively (Table 6).

**Table 5:** ROI analysis results for the win domain (df = 47)

Contrast	*k* threshold*	Contrast direction	Region	Laterality	Peak MNI coordinates			Cluster size (*k*)	Peak *t*
					*x*	*y*	*z*		
RP > 0	34	Positive	AI	Left	n.s.	n.s.	n.s.		
				Right					
			vmPFC	Left	−9	47	−13	556	4.60
				Right	6	50	−10	556	4.80
			VS	Left	n.s.	n.s.	n.s.		
				Right					
		Negative	AI	Left	−33	23	5	105	5.26
				Right	n.s.	n.s.	n.s.		
			vmPFC	Left	n.s.	n.s.	n.s.		
				Right					
			VS	Left	n.s.	n.s.	n.s.		
				Right					
RPE > 0	32	Positive	AI	Left	−33	17	−7	371	5.29
				Right	30	20	−7	697	7.37
			vmPFC	Left	−3	47	5	583	6.34
				Right	3	47	−1	583	6.35
			VS	Left	−12	5	−10	371	7.95
				Right	12	5	−10	697	7.84
		Negative	AI	Left	n.s.	n.s.	n.s.		
				Right					
			vmPFC	Left	n.s.	n.s.	n.s.		
				Right					
			VS	Left	n.s.	n.s.	n.s.		
				Right					

*Whole-brain corrected, *p* < 0.05 (based on 3dClustSim correction: *k* > 33, *p* < 0.005). n.s., Not significant.

**Table 6:** ROI analysis results for the loss domain (df = 47)

Contrast	*k* threshold*	Contrast direction	Region	Laterality	Peak MNI coordinates			Cluster size (k)	Peak T
					*x*	*y*	*z*		
LP > 0	34	Positive	AI	Left	n.s.	n.s.	n.s.		
				Right					
			vmPFC	Left	−6	29	−10	52	4.43
				Right	0	26	−16	52	4.49
			VS	Left	n.s.	n.s.	n.s.		
				Right					
		Negative	AI	Left	−30	26	−4	114	3.77
				Right	n.s.	n.s.	n.s.		
			vmPFC	Left	n.s.	n.s.	n.s.		
				Right					
			VS	Left	−18	11	−1	114	6.60
				Right	12	8	5	95	5.26
LPE > 0	33	Positive	AI	Left	−30	26	2	47	4.61
				Right	33	29	−1	74	4.26
			vmPFC	Left	n.s.	n.s.	n.s.		
				Right					
			VS	Left	n.s.	n.s.	n.s.		
				Right					
		Negative	AI	Left	n.s.	n.s.	n.s.		
				Right					
			vmPFC	Left	−12	47	−4	390	3.86
				Right	6	41	−13	390	5.58
			VS	Left	−15	11	−10	733	7.95
				Right	24	−1	−13	178	5.26

*Whole-brain corrected *p* < 0.05 (based on 3dClustSim correction: *k* > 33, *p* < 0.005).

### Eye tracking

Analysis of the eye-tracking data revealed that subjects differed neither in the number of total fixations in both domains, nor in fixation differences between values and probabilities (Table 7). However, subjects paid slightly more attention to values compared with probabilities in both the win (one-sample *t* test; mean, 2.51 ± 4.959; *p* = 0.002; df = 43) and the loss domain (one-sample *t* test; mean, 2.73 ± 4.521; *p* < 0.001; df = 43). The two variables Df win and Df loss were highly correlated (Table 8), and the participants did not show differences in fixations on high-risk and low-risk gambles between the gain and loss domains (Table 7). The behavioral results of the eye-tracking task revealed that in both domains subjects made high-risk choices slightly more often than low-risk choices, and that the average reaction time did not differ with regard to domain or choice type (Table 7).

**Table 7:** Descriptive overview of the eye-tracking task variables

Variable	*N*	Minimum	Maximum	Mean	SD
Total fixations, win domain	44	7.90	65.23	29.32	12.726
Total fixations, loss domain	44	7.50	61.17	28.94	11.720
Df win domain: [*f*(*v*) − *f*(*p*)]	44	−9.13	12.20	2.51	4.959
Df loss domain: [*f*(*v*) − *f*(*p*)]	44	−7.77	11.07	2.73	4.521
Df high risk, win domain: [*f*(*h*) − *f*(*l*)]	44	−5.00	2.07	−0.35	1.395
Df high risk, loss domain: [*f*(*h*) − *f*(*l*)]	44	−3.97	4.37	0.34	1.495
Percentage of high-risk choices, win domain	44	26.67	100.00	66.59	20.466
Percentage of high-risk choices, loss domain	44	30.00	100.00	77.27	18.584
Average reaction time, all trials	44	3.02	17.99	8.51	3.544
Average reaction time, win domain	44	2.92	21.63	8.68	3.935
Average reaction time, win domain, high-risk choices	44	2.92	22.68	8.74	3.958
Average reaction time, win domain, low-risk choices	41	3.23	19.76	8.96	4.058
Average reaction time, loss domain	44	3.12	16.38	8.56	3.471
Average reaction time, loss domain, high-risk choices	44	3.12	15.59	8.37	3.363
Average reaction time, loss domain, low-risk choices	44	3.57	38.20	10.33	6.309

**Table 8:** Additional significant Pearson correlations (uncorrected) of the variables from the eye-tracking task

Variable 1	Variable 2	*r*	*P*	*N*
Df win	Df loss	0.94	<0.001	44
	Average reaction time, win domain	−0.39	0.009	44
	Average reaction time, win domain, high-risk choices	−0.38	0.011	44
	Average reaction time, win domain, low-risk choices	−0.38	0.016	41
Df loss	Average reaction time, loss domain	−0.46	0.002	44
	Average reaction time, loss domain, high-risk choices	−0.48	0.001	44
	Average reaction time, loss domain, low-risk choices	−0.36	0.023	44
Df high risk win	Percentage of high-risk choices, win domain	0.31	0.039	44

In both domains, higher reaction times correlated with more fixations on probabilities compared with values (Table 8). Additionally, more fixations on high-risk gambles compared with low-risk gambles correlated with more high-risk gamble decisions, but only in the win domain (Table 8).

### Correlation of fMRI and eye tracking

Bootstrapping the results of the fMRI and eye-tracking data correlations showed that higher activation in the bilateral vmPFC during reward prediction error (RPE > 0) processing correlated with a higher number of fixations on values versus probabilities in the eye-tracking task (Table 9). In the loss domain, left VS activation during loss prediction error (LPE > 0) processing correlated with more fixations on probabilities versus values (Table 9).

**Table 9:** Main bootstrapped Pearson correlation results (uncorrected, seed set at 10 with 10,000 repetitions) of the fMRI and eye-tracking data (df = 42)

Eye-tracking variable	ROI	fMRI contrast variable	
		RP > 0	RPE > 0
Df win	Left AI	−0.05 (0.169)	0.14 (0.142)
	Right AI	0.11 (0.122)	−0.02 (0.145)
	Left OSS	−0.03 (0.189)	0.01 (0.125)
	Right OSS	−0.08 (0.210)	0.02 (0.148)
	PCC	0.11 (0.151)	0.10 (0.129)
	Left vmPFC	0.22 (0.156)	0.31 (0.150)*
	Right vmPFC	0.18 (0.150)	0.40 (0.138)*
	Left VS	0.09 (0.135)	0.23 (0.140)
	Right VS	0.17 (0.140)	0.02 (0.131)
		LP > 0	LPE > 0
Df loss	Left AI	0.10 (0.144)	−0.20 (0.127)
	Right AI	0.13 (0.116)	−0.12 (0.151)
	Left OSS	0.17 (0.129)	−0.12 (0.145)
	Right OSS	0.03 (0.157)	−0.21 (0.142)
	PCC	−0.02 (0.129)	−0.28 (0.134)*
	Left vmPFC	−0.16 (0.159)	−0.17 (0.143)
	Right vmPFC	0.01 (0.156)	−0.15 (0.152)
	Left VS	−0.01 (0.152)	−0.32 (0.152)*
	Right VS	−0.08 (0.163)	<0.01 (0.138)

*p < 0.05.

**Figure 4.**
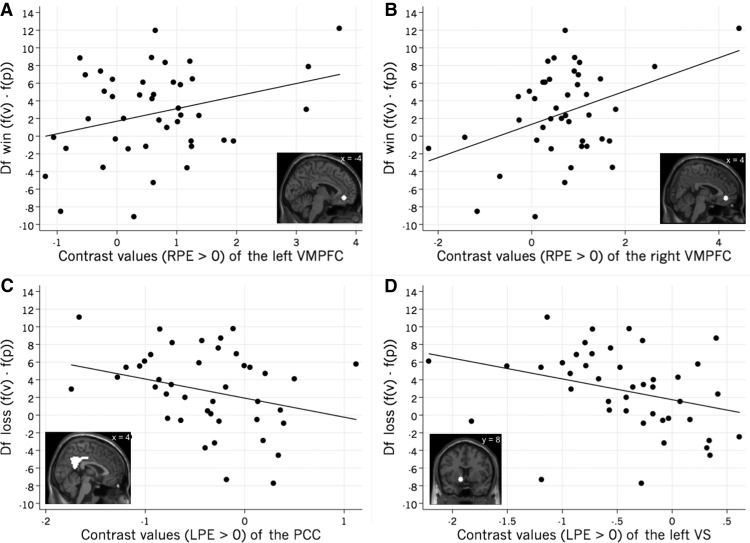
Correlation of fMRI and eye-tracking data, showing individual data points and the respective ROIs used for the contrast value extraction. ***A***, Contrast values of the left VMPFC during RPE processing correlate with higher values of Df win [i.e., increased attention toward values (*f*[*v*]) compared with probabilities (*f*[*p*]) in the win domain; *r* = 0.306, *p* = 0.046, df = 42]. ***B***, Contrast values of the right VMPFC during RPE processing correlate with an increased attention toward values [*f*(*v*)] compared with probabilities [*f*(*p*)] in the win domain (*r* = 0.397, *p* = 0.008, df = 42). ***C***, Contrast values of the PCC during LPE processing correlate with an increased attention toward probabilities [*f*(*p*)] compared with values [*f*(*v*)] in the loss domain (*r* = −0.284, *p*= 0.065, df = 42). ***D***, Contrast values of the left VS during LPE processing correlate with an increased attention toward probabilities [*f*(*p*)] compared with values [*f*(*v*)] in the loss domain (*r* = −0.316, *p* = 0.039, df = 42).

Additionally, PCC activation during LPE processing was found to correlate with decreased Df loss (i.e. increased attention toward probabilities [*f*(*p*)] compared with values [*f*(*v*)] in the loss domain (Table 9). Neither the AI nor the control region OSS correlated significantly with fixation differences in any of the two domains (Table 9). Plotting the correlations revealed individual differences in attention and brain activation (Fig. 4).

## Discussion

We show that individual differences in neural reactions to gains and losses relate significantly to differences in information search over risky gambles. Activity in the vmPFC during gain processing correlated positively with attention to the monetary amounts of risky gambles in the gain domain, while activity in the VS and PCC during loss processing correlated positively with subjects’ attention to the probabilities of gambles in the loss domain. Our results concur with previous findings in the reward domain, in which the vmPFC, VS, and PCC were identified as regions involved in reward and loss processing, and crucially important for value computations and salience (Fliessbach et al., 2007; Seymour et al., 2007; Tom et al., 2007; Bartra et al., 2013; Clithero and Rangel, 2014).

There are a number of studies with a wide array of topics that have previously successfully combined eye tracking and fMRI (Kang et al., 2012; Paschke et al., 2012; Tylén et al., 2012; Meyhöfer et al., 2015). The study of highest relevance for this experiment investigated the relation of attention to value signals (Lim et al., 2011). In the experiment, participants first performed a liking–rating task, which was followed by a binary choice task, during which the subjects were instructed to fixate on the food item and were then asked to make a selection for one of the two food items shown (Lim et al., 2011). During both tasks, fMRI and eye-tracking data were simultaneously recorded, and the researchers found out that activation in the vmPFC and the VS represented a relative value code between the items, which was in turn guided by visual attention measured via eye tracking (Lim et al., 2011). It is important to note here that despite the fact that these findings somewhat overlap with our results, there has been neither a study that has combined eye-tracking and fMRI data collected in two separate experiments, nor a study that has investigated the neural relations of attention patterns in a monetary gambling task. We henceforth add to previous studies by relating individual differences in neural reactions to gains and losses to differences in information search processes, as measured by an independent eye-tracking experiment using risky gambles.

Individual brain activation differences have previously been shown to correlate with behavioral risk preferences. Specifically, risk averters were shown to exhibit higher VS and AI activation during high-risk gamble anticipation (Rudorf et al., 2012). Further correlations of individual brain activation differences using two separate assessment techniques have been described using the behavioral inhibition scale (BIS) and behaviorial activation scale (BAS), and an fMRI task involving monetary gains and losses (Kim et al., 2015). People with higher BIS scores exhibited higher activation of the left striatum during avoidance anticipation, while individuals with higher BAS scores showed higher activation of the bilateral striatum during reward reception. In the eye-tracking domain, individual differences of attention were previously shown in a study involving similar financial gambles (Fiedler and Glöckner, 2012). With these individual differences having previously been found using fMRI or eye tracking, our study is the first to show their relation in a financial decision-making context and is thus able to add an important piece to how attention and value computations are linked.

We have to note obviously that the exclusion of a controlled intervention in our study design inherently limits causal conclusions. Hence, one interpretation is that paying more attention to either monetary amounts or probabilities may cause differences in activation in the vmPFC, VS, and PCC. This hypothesis is supported by previous studies, which showed that exogenous manipulation of attention can successfully change brain activation patterns and ultimately influence behavioral choices, as shown, for example, using cues in the context of food choices (Hare et al., 2011a; Milosavljevic et al., 2012). In an eye-tracking experiment implementing everyday supermarket decisions, manipulation of product salience led to participants making incoherent, “wrong,” product choices, with individuals displaying a visual saliency bias and thereby depicting the strong influence of attention manipulation on decision-making (Milosavljevic et al., 2012). Furthermore, results from a behavioral binary-choice experiment using electroencephalography showed that the additional presentation of a value-associated distractor was associated with making more incorrect decisions and differences in the P300 brain activation amplitude (Itthipuripat et al., 2015).

Another interpretation of the detected correlation is that the vmPFC acts as a driver and guides attention during the information search in risky gambles. This can be placed into the context of a very recent model by Schultz (2016). He describes reward as having inherent sensory and value components, thus leading from object detection to its identification; from there to its valuation; and finally to the decision, action, and reinforcement. Placing our results into the context of this model, the vmPFC can be described as a possible modulator guiding object detection, identification, and valuation. The VS has previously been identified to play a role in risk and prediction error computation, thus leading to the idea that already during the information search phase, the VS could be involved in the perception of risks by guiding information search processes. The laterality of only the left VS being significantly associated with attention patterns is surprising, considering that Lim et al. (2011) found the bilateral VS to be associated with attention, Smith et al. (2014) associated activation from a bilateral VS mask with success in a stock exchange paradigm, and both the left and the right VS have been associated in a meta-analysis investigating the neural basis of subjective value (Clithero and Rangel, 2014). Since reward and loss processing in our study was found in the bilateral VS as well, we are not able to systematically determine why this laterality occurred. Previous studies have investigated such laterality differences in the dopamine response to reward in the VS (Martin-Soelch et al., 2011), but the decision-making field is lacking a comprehensive meta-analysis of especially VS laterality in reward and loss processing, which would greatly help the understanding of such incidental findings.

We did not expect a priori that the PCC would also be associated with attention patterns during risky choices. Finding this correlation, however, points toward the PCC playing an important role in guiding attention more toward the probabilities of risky gambles compared with their values in the loss condition. Notably, the PCC cluster used in our analysis included the location of the PCC previously shown to be associated with attention toward relative value (Lim et al., 2011). Additionally, a meta-analysis by Clithero and Rangel (2014) found that both ventral and dorsal areas of the PCC (both included in the cluster used here) were associated with processing value during the decision phase. A review article by Leech and Sharp (2014), investigating the role of the PCC in relation to disease and cognition, sheds even more light onto our findings. Besides showing the role of the PCC in regulating the focus of attention that concurred with our findings, the authors also introduce an “Arousal, Balance, and Breadth of Attention” (ABBA) model pertaining to the PCC. This model highlights the sensitivity of the PCC to arousal, internal/external thought, as well as attentional focus. In light of this model, the individual differences seen in our PCC results can be interpreted as brain activation related to these dimensions. Another study on the PCC (Studer et al., 2015) presented evidence that lesions in the PCC are detrimental to optimal risky decision-making. Importantly, PCC damage was inversely related to risk adjustment, thus showing the importance of the PCC in risky decision-making. Taking our findings and the previous literature into account, we believe that the role of the PCC in financial decision-making has been somewhat underestimated in the past and should be further investigated in future studies of financial decision-making.

Despite our data not being able to determine causality, we believe that our results are essential to advance the knowledge of decision-making processes, and to lay the basis for future decision-making studies that are aiming to combine fMRI and eye-tracking data in a behavioral economic context. Leaving the causality discussion aside, the correlation depicted by our data can also be seen as evidence for the previously noted bidirectional relationship of eye movements and decision-making (Gottlieb et al., 2014). This relationship is described as values of different environmental stimuli influencing eye movements, with eye movements in turn influencing decision-making due to selecting the sensory input that impacts a person’s decision. In this context, our results provide evidence that the bilateral vmPFC, left VS, and PCC represent three regions responsible for this interaction, henceforth playing a very important role in financial decision-making involving risks. Despite finding these results, a lack of a significant correlation between activation in the AI and attention patterns was noted as well. A reason for this might be that the fMRI paradigm did not specifically elicit brain activation related to risk, but in relation to reward and loss processing. It could therefore be of interest in future studies to check whether the AI activation is related to attention patterns in a risk-specific fMRI paradigm.

Peak activation in the VS was highly correlated to the RPE parameter, a finding that concurs well with previous discoveries (Schultz and Dickinson, 2000; Hare et al., 2008, 2011b; Fliessbach et al., 2010; Glimcher, 2011; Rohe et al., 2012; Häusler et al., 2015). As hypothesized based on the findings of previous studies (Kuhnen and Knutson, 2005; Izuma et al., 2008; Lin et al., 2012), activation of the vmPFC during all parts of reward processing was observed. Besides the vmPFC, other areas were found to be activated during both reward prediction and reward prediction error processing. This could have been the case because the reward prediction phase can also be seen as a time point of reward prediction error processing. The cue shown during reward prediction can elicit a prediction error response in that the probability of possible wins or losses may deviate from previous predictions.

With regard to the eye-tracking findings, the high correlation of the fixation differences in both domains (Df win and Df loss) shows the attention consistency of individuals during the information search phase, irrespective of being in a win or loss context. We observe a rather risk-seeking behavior in our experiment, possibly due to the fact that the gambles were performed with money that was additionally earned on top of the “safe” participation fee. It could be of interest to run the same experiment again without such a participation fee, thus having the subjects complete the eye-tracking experiment without a safe payment in the back of their mind. Finally, more fixations on high-risk compared with low-risk gambles correlated with more high-risk gamble decisions. This concurs with previous findings identifying choice as a function of attention (Brandstätter and Körner, 2014).

By combining fMRI with eye tracking using two separate tasks, our study shows that information search in risky gambles is related to neural reward and loss processing in the vmPFC, VS, and PCC. Higher reward processing activity in the vmPFC was correlated to paying more attention to the monetary values compared with their respective probabilities in risky gambles. Additionally, higher loss processing activity in the left VS and the PCC was correlated to paying more attention to the probabilities compared with the monetary values of risky gambles. Future studies will need to dig deeper into the causality of this link.

## Synthesis

The decision was a result of the Reviewing Editor Daniela Schiller and the peer reviewers coming together and discussing their recommendations until a consensus was reached. A fact-based synthesis statement explaining their decision and outlining what is needed to prepare a revision is listed below. The following reviewers agreed to reveal their identity: Gregory Samanez-Larkin

This is an interesting study that combines several different pieces of data. Although the authors have addressed some of the previous concerns, there are several remaining issues, particularly the interpretation of the GLMs and the specificity of the effects; there are also some new questions for the authors.

1) The categorical win>nowin and continuous PE regressors are pretty much the same thing except that the continuous one is likely more sensitive. It's still not clear to me why the categorical one is necessary but it should be made clear that the only difference between them is that one is categorical and one is continuous. If the authors believe that there is some other interesting signal that you get from the categorical analysis that is not PE-related, that should also be made more explicit.

2) Phrases like this imply that there is selectivity in the effects: "Participants with higher gain-related activity pay more attention to the value of risky gambles, while subjects showing higher loss-related activity pay more attention to the associated probabilities". Table 9 needs to include all of the correlations that were run so that the specificity (or lack of) is made clearer. Even if the effects are not that specific, the paper is still interesting. It's just best to have all of this on display for readers to evaluate, and the corresponding discussion should reflect the described results.

3) Figure 3 is confusing - it is discussed as results from ROI analyses, but the maps seem to be whole-brain results. For example, there is clearly a large PCC area of significance in the top left corner. Please clarify.

4) Related to the second point, why is PCC not explored as well? It would seem possible that correlations like those found for VMPFC and VSTR might be found in PCC, too. This could also complement some of the discussions of PCC in the meta-analyses that the authors list.

5) The bottom middle figure in Figure 3 for VSTR seems to not show a significant result, but then there is a significant correlation shown in Figure 4. Please add more details and clarify.

6) Are there no significant results (Figure 4, Table 9) for right VSTR? It would be good for the authors to discuss why it might be only left VSTR. How were the button configurations set up for the task and responses? Perhaps this means a subset of fMRI trials have a stronger attentional effect than others?

7) It seems a bit strange to have not considered other neuroeconomic studies that do combine fMRI with eye-tracking, collected in the scanner. The most prominent one - although there are others - is Lim, O'Doherty, Rangel (2011). There is definitely some overlap in findings, but it would be nice to incorporate the Lim (2011) results into the current discussion.

8) In the fMRI model cue-related signals are called "prediction" and outcome signals are called "prediction errors", but it is important to note that these are all prediction errors.
